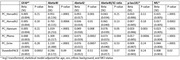# Association of Epigenetic Aging with Plasma Biomarkers of Amyloid, Tau, Neurodegeneration and Neuroinflammation in Diverse Hispanic/Latino Adults

**DOI:** 10.1002/alz.093265

**Published:** 2025-01-09

**Authors:** Hector González, Wassim Tarraf, Kevin A Gonzalez, Sayaka Kuwayama, Humberto Parada, Alberto R Ramos, Tatjana Rundek, Krista M Perreira, Bonnie Levin, Charles Decarli, Myriam Fornage

**Affiliations:** ^1^ UCSD, La Jolla, CA USA; ^2^ Wayne State University, Detroit, MI USA; ^3^ University of California, San Diego, La Jolla, CA USA; ^4^ San Diego State University, San Diego, CA USA; ^5^ University of Miami Miller School of Medicine, Miami, FL USA; ^6^ Evelyn F. McKnight Brain Institute, Miami, FL USA; ^7^ University of North Carolina, Chapel Hill, NC USA; ^8^ University of California, Davis, Davis, CA USA; ^9^ Human Genetics Center, School of Public Health, University of Texas Health Science Center, Houston, TX USA; ^10^ The University of Texas Health Science Center at Houston, Houston, TX USA

## Abstract

**Background:**

Epigenetic clocks are biomarkers of biological age based on DNA methylation (DNAm) patterns and are widely used as predictors of health and aging outcomes. Multiple epigenetic clocks have been developed and reflect different aspects of the multidimensional aging process, above and beyond chronological age. To date, no study has examined the relationship of epigenetic aging with circulating biomarkers of Alzheimer’s Disease (AD). We investigated the association of 6 epigenetic clocks with plasma glial fibrillary acidic protein (GFAP), amyloid beta 40 and 42, phosphorylated tau 181 (p‐tau181), and neurofilament light chain (NfL) in diverse Hispanic/Latino adults.

**Method:**

We estimated PC‐based epigenetic age acceleration from 6 epigenetic clocks in 2662 Hispanic and Latino adults (mean age 64.1 years; 56% women) from the Hispanic Community Health Study/Study of Latinos (HCHS/SOL) who had available blood DNAm and plasma biomarker data at visit 2. These included first generation clocks, Horvath1, Horvath2 (“skin‐blood”) and Hannum clocks; second generation PhenoAge and GrimAge; and third generation DunedinPACE. We used survey linear regression weighted least squares to estimate the association of each of these measures with each of the plasma biomarkers.

**Result:**

There were varying strengths of association between the clocks and the plasma biomarkers. There were significant associations of faster acceleration in all epigenetic clocks with higher plasma levels of NfL. There were significant associations of faster acceleration in all epigenetic clocks except DunedinPACE with higher plasma levels of Abeta40 and Abeta42. Faster PhenoAge acceleration was associated with higher levels of all circulating biomarkers (Table).

**Conclusion:**

Epigenetic age acceleration is associated with circulating biomarkers of amyloid, tau, neurodegeneration and neuroinflammation in diverse Hispanics/Latinos. The second‐generation clock PhenoAge, trained on nine clinical biomarkers and chronological age, showed consistent and strongest associations across all biomarkers, suggesting that it may be particularly relevant to predicting AD.